# Matrix Type Influences the Levels of Soluble Immune Checkpoints

**DOI:** 10.1002/jcla.70153

**Published:** 2025-12-29

**Authors:** Veronica Buia, Martina Bonacini, Cecilia Catellani, Alessandro Rossi, Francesco Muratore, Carlo Salvarani, Alessandro Zerbini, Stefania Croci

**Affiliations:** ^1^ PhD Program in Clinical and Experimental Medicine, University of Modena and Reggio Emilia Modena Italy; ^2^ Unit of Clinical Immunology, Allergy and Advanced Biotechnologies, AUSL – IRCCS di Reggio Emilia Italy; ^3^ Unit of Rheumatology, AUSL ‐ IRCCS di Reggio Emilia Italy; ^4^ Department of Surgery, Medicine Dentistry and Morphological Sciences With Interest in Transplant University of Modena and Reggio Emilia Modena Italy

**Keywords:** cancer, immune‐mediated diseases, plasma, serum, soluble immune checkpoints

## Abstract

**Background:**

Soluble immune checkpoints (sICs) are emerging as possible serum and plasma biomarkers in cancer and immune‐mediated diseases, but little is known about the impact of the matrix type in sIC detection. This study aimed to assess whether sIC measurements are comparable between serum and EDTA‐plasma samples.

**Methods:**

A cohort of 38 healthy subjects was enrolled. A multiplex bead‐based assay was used to evaluate a panel of 17 sICs (CD137, 4‐1BBL, CD27, CTLA4/CD152, CD80, CD40, CD40L, GITR, GITRL, ICOSL, IDO, LAG3, PD‐1, PD‐L1, PD‐L2, TIM3, and VISTA) in paired serum and plasma‐EDTA samples. The detection frequencies, concentrations, and correlations of each sIC were analyzed by comparing the two matrices.

**Results:**

Soluble CD137, CD152, CD40, and LAG3 were detected more frequently in plasma, while soluble CD40L was detected predominantly in serum. The concentrations of soluble 4‐1BBL, CD27, PD‐1, VISTA were higher in plasma, while the concentrations of soluble PD‐L2 were higher in serum. The concentrations of soluble CD80, GITR, GITRL, ICOSL, IDO, and TIM3 were comparable between serum and plasma. Soluble CD27, CD80, GITRL showed a significant positive, slight correlation between plasmatic and serum concentrations.

**Conclusion:**

Except for soluble CD80, the detection of the other sICs by the bead‐based assay was influenced by the matrix type. The evaluation of the best matrix for sICs should be considered before starting clinical studies.

## Graphical abstract

This study aimed to assess whether the quantification of soluble immune checkpoints (sICs) is comparable between serum and EDTA‐plasma samples. We quantified 17 sICs by a multiplex bead‐based assay in paired serum and plasma‐EDTA samples collected from a cohort of 38 healthy subjects. The frequencies of detection, concentrations, and correlations of each sICs were analyzed. Except for soluble CD80, the detection of the other sICs was influenced by the matrix type, underscoring the importance of the matrix when interpreting sIC results.

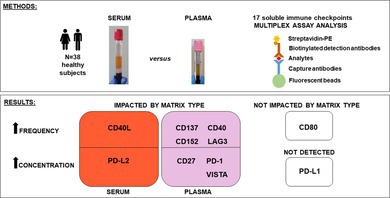

## 1 Introduction

Immune checkpoints (ICs) consist of paired receptor‐ligand molecules with either stimulatory or inhibitory effects on immune responses, including immune surveillance and regulation [1]. They are expressed as plasma membrane proteins (mICs) and can be found on various cell types, including monocytes, NK cells, T cells, dendritic cells, stromal cells, antigen‐presenting cells, and tumor cells [1, 2]. In addition to the mICs, soluble forms (sICs) of both receptors and ligands have also been identified. They can be generated through enzymatic cleavage of the mICs mediated by metalloproteinase or by alternative splicing of mRNA [3, 4]. Although mICs have been largely explored in cancer immunity and immunotherapy, the biological and clinical significance of soluble forms remains poorly understood. These soluble forms have emerged as potential prognostic biomarkers of disease, particularly in predicting outcomes and treatment responses in various types of tumors [5]. For example, serum soluble PD‐L1 levels at diagnosis were associated with overall survival in urinary bladder cancer patients [6]. Furthermore, in glioma patients' serum, soluble PD‐L1 levels correlated with disease grade [7, 8]. Serum soluble PD‐1 and PD‐L1 emerged also as potential biomarkers for risk stratification in patients with classical Hodgkin lymphoma [9]. While, plasmatic soluble PD‐L1 and PD‐1 levels were predictors of response to neoadjuvant chemotherapy in breast cancer patients [10]. In addition to blood (serum and plasma), other matrices, including urine, synovial fluids, cerebrospinal fluids, and peritoneal fluids, have also been explored for sIC detection, although their utility is still under investigation [11, 12, 13, 14, 15].

It is known that the quantification of soluble analytes can be influenced by the choice of matrix type [16]. The specific characteristics of each matrix can affect the quantification of biomarkers, and differences between serum and plasma may lead to discordant results for the same analytes [17]. Given their emerging role as disease biomarkers, investigating the impact of matrix type on sIC detection is important for clinical practice. To date, the comparison among the same sICs in serum and plasma within the same cohort is poorly investigated, and few data are available only regarding soluble PD‐L1 and soluble CD40L [17, 18]. Therefore, the aim of this study is to determine whether serum and plasma provide comparable measurements of sICs.

## 2 Materials and Methods

### 2.1 Cohort of Subjects

A cohort of 38 healthy subjects was recruited at the Azienda Unità Sanitaria Locale—IRCCS of Reggio Emilia, Italy. Inclusion criteria were: age > 18 years; absence of a previous history of cancer; absence of diabetes; no pregnancy; written informed consent. The median age was 60.5 years (interquartile range; IQR: 39.5–72.0), and gender distribution was: 73.68% female (28/38) and 26.32% male (10/38).

### 2.2 Biological Samples

A venous blood sample was drawn from each healthy subject and collected into EDTA‐coated tubes (BD, ref.: 367525) K2EDTA (1,8 mg/mL) and in serum gel separator tubes (BD, Vacutainer SST tubes ref.: 362076). To obtain serum sample, peripheral blood was stored at room temperature for at least 30 min to reach full coagulation. Plasma and serum samples were obtained by centrifugation at 1800 × *g* for 20 min. Samples were aliquoted and stored at −80°C until use.

### 2.3 sIC Quantification

A custom multiplex bead‐based assay validated for both serum and plasma matrix (ProcartaPlex 17‐plex kit) was used to simultaneously quantify the serum and plasma concentrations of the following sICs: tumor necrosis factor receptor superfamily member 9 (TNFRSF9/CD137), its ligand (TNFSF9/4‐1BBL), CD27, cytotoxic T‐lymphocyte associated protein 4 (CTLA4/CD152), CD80, CD40 and its ligand CD40L, glucocorticoid‐induced TNFR‐related protein (GITR) and its ligand GITRL, inducible costimulator‐ligand (ICOSL), idoleamine‐pyrrole 2,3‐dioxygenase (IDO), lymphocyte‐activation gene 3 (LAG3), programmed cell death protein 1 (PD‐1) and its ligands PD‐L1 and PD‐L2, T‐cell immunoglobulin and mucin‐domain containing‐3 (TIM3), and V‐domain Ig suppressor of T cell activation (VISTA). Serum and plasma samples were thawed, centrifuged at 10,000 × *g* for 5 min at room temperature, and diluted in universal assay buffer 1:2 to reduce potential matrix effects. Standards were also reconstituted in universal assay buffer as suggested by the manufacturers' instruction and eight 1:4 serial dilutions of standards plus blank were included. Serum and plasma from the same subjects were tested in the same assay.

The Bio‐Plex MAGPIX Multiplex Reader instrument was used for data acquisition, with a lower bound of 100 beads per sample per sIC. The data were processed by Bio‐Plex Manager MP software (Bio‐Rad, Hercules, CA, USA). Standard curves were calculated with an asymmetric sigmoidal, five‐parameter logistic equation. As upper and lower detection limits (UDLs, LDLs) we considered the most concentrated and diluted standards used to generate the standard curves of the various sICs. The detection limits for each sIC are reported in Table S1.

### 2.4 Statistical Analysis

Statistical analysis was performed using GraphPad Prism 10 software. Heatmap was designed to show the presence or absence of soluble immune checkpoints detected simultaneously in serum and plasma samples, and Fisher's exact test was used to compare the frequencies of detection. To compare the concentrations of sIC in the two matrices, a non‐parametric Wilcoxon matched‐pairs signed rank test was used. Spearman's correlation analysis was performed to evaluate correlations between quantitative variables. Values below the LDLs were considered as “not detected”. To perform the analyzes values = 1/4 of the LDLs were arbitrarily assigned. *p* values < 0.05 (two‐tailed) were considered statistically significant.

## 3 Results

### 3.1 Frequency of Detection of sICs in Serum and Plasma

**FIGURE 1:**
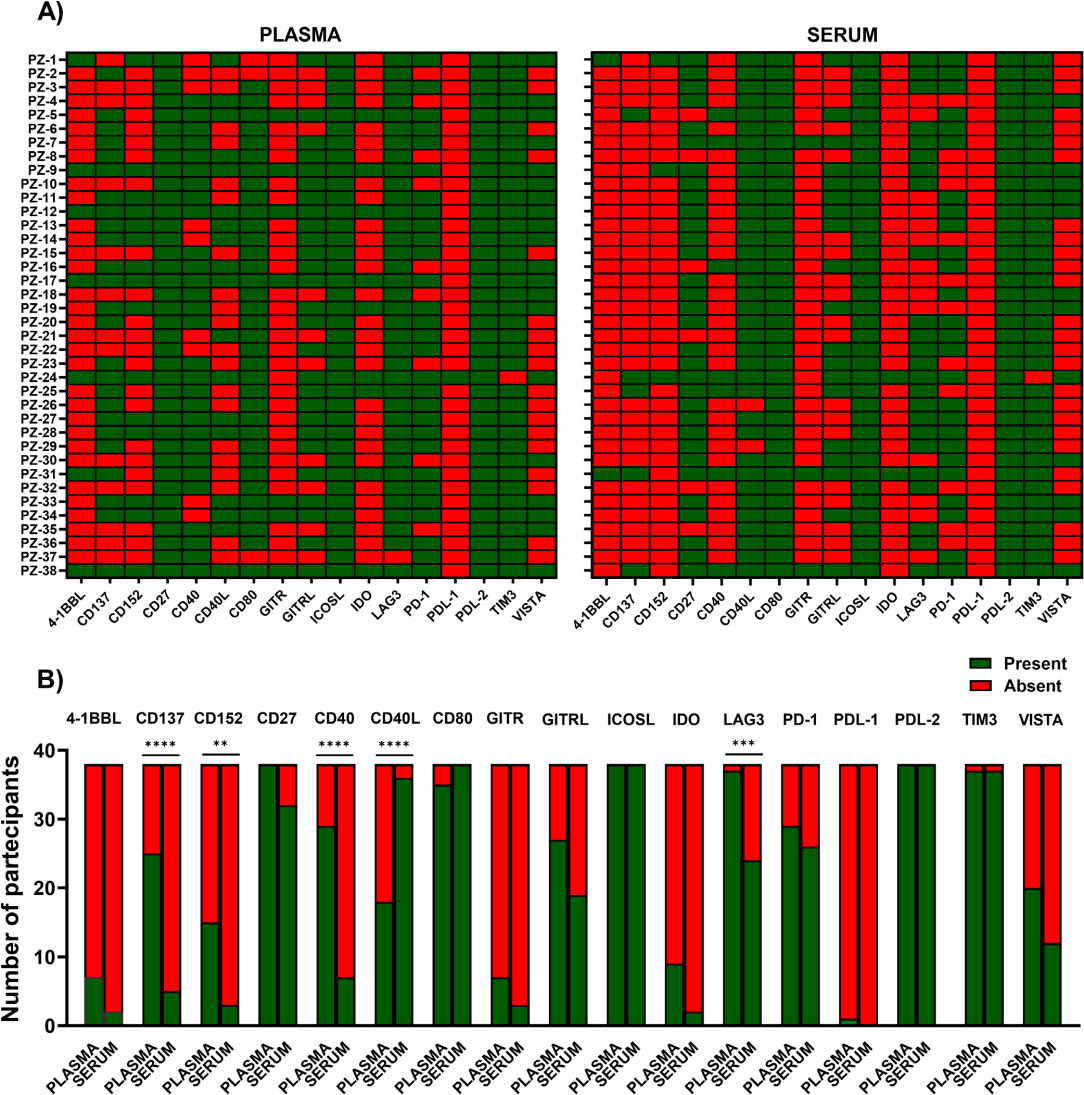
Detection of sICs in plasma and serum samples. (A) Heatmaps of the presence/absence of sICs in the two matrices for each subject (*n* = 38). The absent sICs are reported in red, while those present in green. (B) Histograms of the sICs' frequency of detection in plasma and serum. Data were analyzed with Fisher's exact test and *p* < 0.05 were considered statistically significant. *p < 0.05; **p < 0.01; ***p < 0.001; ****p < 0.0001.

Seventeen sICs were simultaneously evaluated in serum and plasma samples from a cohort of 38 healthy subjects. Heatmaps were generated to illustrate the presence or absence of each sIC, comparing plasma and serum samples and to highlight possible differences between these two matrices (Figure 1A). Notably, soluble ICOSL, PD‐L2, and TIM3, with the exception of only one sample, were detected in all the tested samples in both plasma and serum matrices, whereas soluble PD‐L1, with the exception of one sample, was not detected. Soluble CD27 was found in all plasma samples, whereas soluble CD80 was found in all serum samples. Since the other sICs were present in a fraction of the samples, we used Fisher's test to evaluate potential differential frequencies of detection. The analysis revealed that soluble CD137 (*p* < 0.0001), CD152 (*p* = 0.0024), CD40 (*p* < 0.0001), and LAG3 (*p* = 0.0003) were more frequently detected in plasma compared to serum, while soluble CD40L (*p* < 0.0001) was predominantly detected in serum (Figure 1B). Furthermore, no statistically significant differences in the frequencies of detection were observed for soluble 4‐1BBL, CD27, CD80, GITR, GITRL, ICOSL, IDO, PD‐1, PDL‐1, PDL‐2, TIM3, and VISTA.

### 3.2 Concentrations of sICs in Serum and Plasma

**FIGURE 2:**
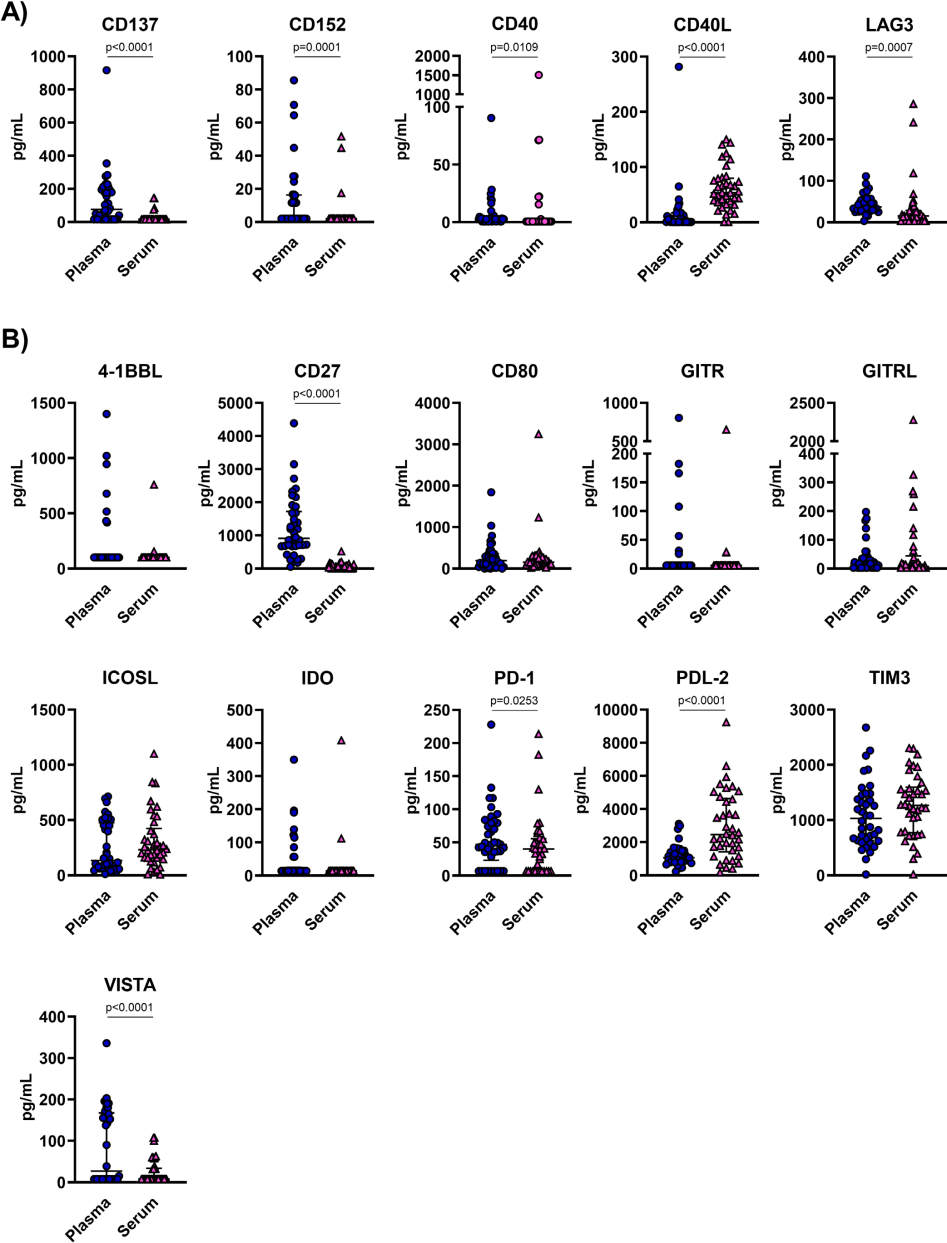
Concentrations of sICs in plasma and serum samples. Concentrations of sICs in plasma (blue dots) and serum (pink triangle) samples. Panel (A) shows the sICs detected predominantly in one matrix type, while the panel (B) illustrates the sICs detected at the same frequency between plasma and serum. Differences between the two groups were analyzed using the Wilcoxon matched‐pairs signed rank test and *p*‐values < 0.05 were considered statistically significant. Concentrations of each sIC are expressed in pg/ml and the median is shown as solid lines in each groups. Regarding sICs whose concentrations were below the LDLs, a value = 1/4 of the most diluted standard was arbitrarily assigned.

To evaluate the differences in sIC levels between serum and plasma samples, we compared the pg/mL of each sIC in the two matrices using the Wilcoxon signed‐rank test. Based on the above results, the molecules were categorized into two groups: sICs detected predominantly in either serum or plasma samples (Figure 2A), and sICs detected at the same frequency (Figure 2B).

Concerning the sICs detected with different frequencies in plasma and serum, we also observed differences in the concentrations detected in the two matrix types. Specifically, soluble CD137, CD152, CD40, and LAG3 concentrations were significantly higher in plasma than in serum samples by 4.8‐, 4.2‐, 4.0‐, and 6.3‐ fold, respectively (Figure 2A). Instead, soluble CD40L concentrations were significantly 46‐fold higher in serum than in plasma samples (Figure 2A).

Regarding the sICs detected with the same frequencies in the two matrices, soluble 4‐1BBL, CD80, GITR, GITRL, ICOSL, IDO, and TIM3 showed comparable concentrations between plasma and serum. Instead, soluble PDL‐2 levels were 2.9‐fold higher in serum (serum median concentration: 2450 pg/mL, IQR: 1421–4626; plasma median concentration: 1063 pg/mL, IQR: 813–1486), whereas soluble CD27, PD‐1, and VISTA levels were more elevated in plasma than serum (CD27 plasma median concentration: 910 pg/mL, IQR: 671–1724; CD27 serum median concentration: 37.1 pg/mL, IQR: 18.0–116.5; PD‐1 plasma median concentration: 47 pg/mL, IQR: 23–80.2; PD‐1 serum median concentration: 40 pg/mL, IQR: 6.8–55.6; VISTA plasma median concentration: 27.1 pg/mL, IQR: 8.0–167.3; VISTA serum median concentration: 8.0 pg/mL, IQR: 8.0–33.9) (Figure 2B).

### 3.3 Correlation of sIC Concentration in the Two Matrices

**FIGURE 3:**
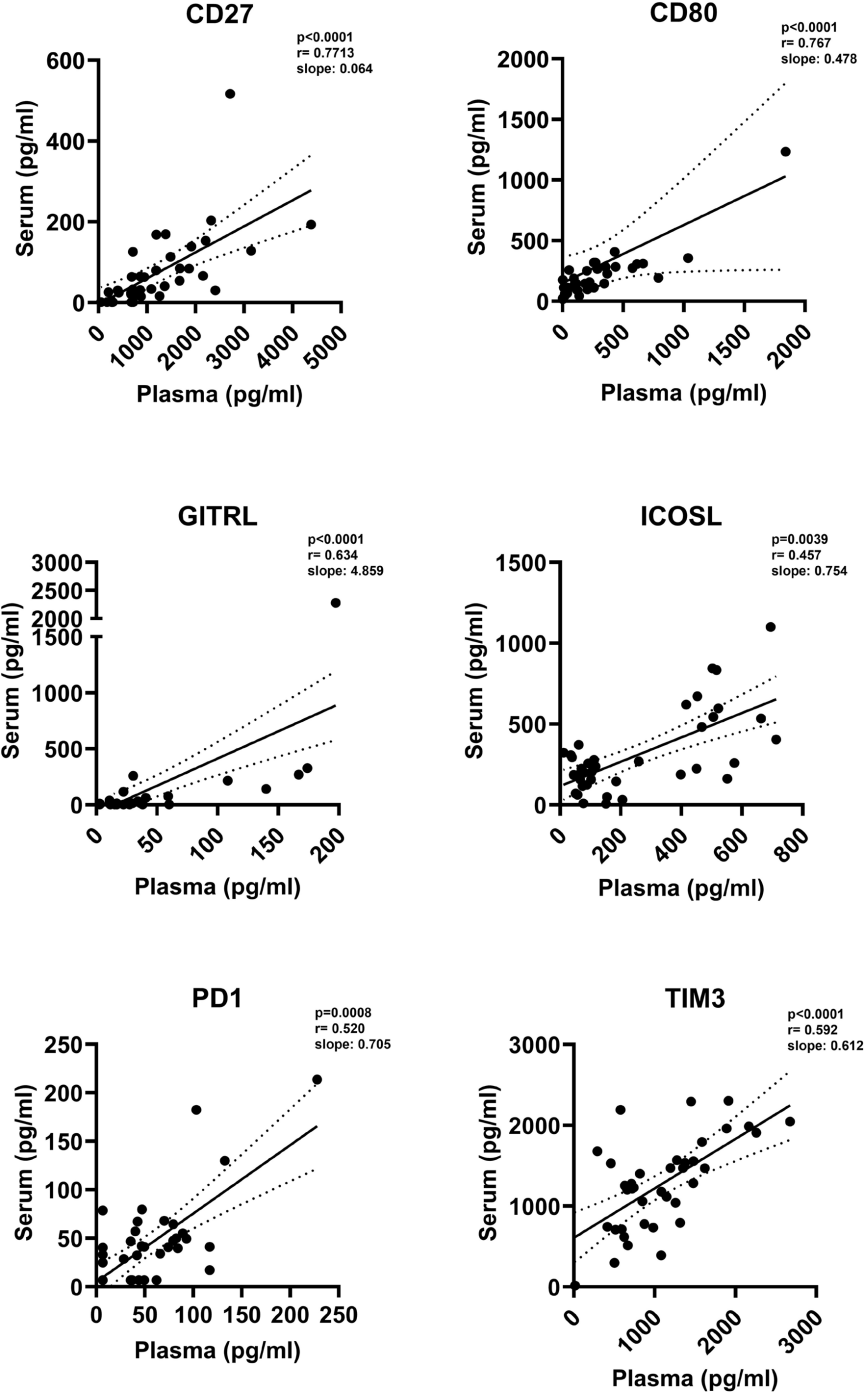
Correlations between plasma and serum levels of sICs. Correlations between serum and plasma concentrations of sICs are depicted. Spearman's correlation test was used and *p*‐values < 0.05 were considered statistically significant. Solid line represents the linear regression of the correlation, while dotted lines represent the standard error.

Finally, we performed a correlation analysis for each sIC to evaluate if data obtained from the two matrix types were comparable. Concerning sICs detected with the same frequency and concentration levels in plasma and serum samples, a weak positive correlation was identified for soluble CD80 and GITRL, ICOSL, and TIM3, even if the correlation coefficients were lower than 0.8. Soluble CD80 showed the best correlation between the two matrices (*r* = 0.767, *p* < 0.0001) (Figure 3). Despite soluble 4‐1BBL, GITR, and IDO being detected at the same frequency and the median concentrations in serum and plasma samples being comparable, we did not observe a correlation of their levels between the two matrix types.

Regarding soluble CD137, CD152, CD40, CD40L, and LAG3, the sICs detected in plasma and serum with differential frequencies; no correlations were observed between the plasmatic and serum concentrations. No correlations were also observed for soluble PD‐L2 and VISTA, the sICs detected at different levels between the two matrices. Conversely, even if soluble CD27 and PD‐1 were higher in plasma than serum, a correlation between plasmatic and serum levels was observed (*r* = 0.7713, *p* < 0.0001 and *r* = 0.520, *p* = 0.0008 respectively, Figure 3). Table 1 summarizes the results on frequencies of detection, concentrations, and correlations for each sIC between serum and plasma.

**TABLE 1:** Summary of differences in frequency, concentration, and correlation for each sIC.

	Frequency	Concentration	Correlation
4‐1BBL	Yes	Yes	No
CD137	No	No	No
CD152	No	No	No
CD27	Yes	No	Yes
CD40	No	No	No
CD40L	No	No	No
CD80	Yes	Yes	Yes
GITR	Yes	Yes	No
GITRL	Yes	Yes	Yes*
ICOSL	Yes	Yes	Yes*
IDO	Yes	Yes	No
LAG3	No	No	No
PD‐1	Yes	No	Yes*
PDL‐1	Yes	Non detected	Not applicable
PDL‐2	Yes	No	No
TIM3	Yes	Yes	Yes*
VISTA	Yes	No	No

*Note:* Soluble ICs with the same frequency, concentration, and positive correlation between serum and plasma are reported as “Yes”; sICs with differences in frequency, concentration, and correlation are reported as “No”. *, weak correlation with *r* < 0.7.

## 4 Discussion

Soluble ICs are emerging as diagnostic and prognostic biomarkers in diseases, and their simultaneous detection across different matrix types could be useful for clinical practice. Human serum and plasma are typical matrices used in clinical and biological studies. It is known that many pre‐analytical factors, including the choice of biological matrix types, can influence the detection of soluble proteins [16, 17, 19, 20, 21, 22, 23, 24]; however, few studies investigated the effects of matrix on sICs [17, 18, 25]. The aim of this study was to determine whether serum and plasma could provide comparable results on sICs in a cohort of healthy subjects. We analyzed 17 sICs in both serum and plasma‐EDTA to investigate possible differences in their frequencies of detection and concentrations and to evaluate their interchangeability. Our findings demonstrate that the matrix type can affect both the detection frequencies and concentrations of specific sICs.

Available data in the literature on sICs are about CD40L, PD‐1, PD‐L1, and PD‐L2 [17, 18, 25, 26], while we demonstrated for the first time that the choice of matrix affects the detection of 4‐1BBL, CD137, CD152, CD27, CD40, GITR, GITRL, ICOSL, IDO, LAG3, TIM3, and VISTA. Concerning soluble CD40L, our results are in line with literature data. Indeed, Wenzel and collaborators showed higher concentrations of soluble CD40L in serum than in plasma‐EDTA and plasma‐citrate from healthy donors [18]. In addition, higher concentrations of soluble CD40L in serum than in plasma‐heparin were also reported by Rosenberg‐Hasson and researchers [16]. Finally, Varo et al. also showed that the concentrations of soluble CD40L were higher in serum than in plasma‐citrate, plasma‐EDTA, and plasma‐heparin from healthy volunteers [25]. To notice, none of these studies considered the frequencies of detection or performed correlation analysis.

To the best of our knowledge, only two studies have compared the concentrations of soluble PD‐L1 in serum and plasma samples, reporting higher levels of soluble PD‐L1 in serum than in plasma [17, 26]. Instead, we did not detect soluble PDL‐1 in both matrices. Differences in the nature of the cohorts of subjects could explain this discrepancy: we analyzed a cohort of healthy subjects while Cappelletto et al. analyzed melanoma and non‐small cell lung cancer patients. Moreover, differences in the assays (e.g., different antibody pairs, methods of detection, composition of binding and wash buffers as well as the detection limits of each assay) could provide different results. Moreover, Krueger et al. reported higher recovery of PD‐1 and PD‐L2 in serum than plasma [26].

Several studies have reported that quantification of cytokines in serum does not always reflect those in plasma [17, 19, 20, 21, 24]. The matrix effect emerged as biomarker‐dependent and subject‐dependent [16, 20]. Moreover, within plasma matrix, different anti‐coagulants used for blood collection could interfere with cytokine detection [19, 24]. Serum samples are obtained after a coagulation process, while anti‐coagulants present in the tubes for plasma collection block the coagulation process. To one hand, during the clot formation, the fibrin molecules cross‐link together. This can trap some circulating proteins into the clot with the consequent reduction of their detection. To the other hand, the coagulation process engages the activation of platelets, which can release soluble mediators. Regarding sICs, it was demonstrated that CD40L and PD‐L1 are produced by activated platelets, explaining the higher levels of these sICs in serum than plasma [25, 27]. Interestingly, Tworoger et al. reported that plasma‐EDTA is the best matrix for proteomic analysis [28]. Our findings indicate that plasma offers better quantification of soluble CD137, CD152, CD40, LAG3, CD27, and VISTA. Our findings indicate that the detection of soluble CD80 is not influenced by matrix type. Regarding soluble GITRL, ICOSL, and TIM3, although they showed comparable frequencies of detection and concentrations, the correlation coefficients between serum and plasma levels were low to exclude matrix effects.

We decided to analyze samples from healthy subjects because the concentrations of analytes in plasma and serum could be disease‐specific/disease‐dependent. The inclusion of a cohort of patients would open the question of which pathology would be most suitable. A methodological comparison between different matrices using healthy subject cohorts represents the first step before studying the possible clinical relevance of sICs in specific diseases.

The limit of the present study is that we did not cross‐validate the results using multiplex kits from different companies or using different platforms to compare assay‐to‐assay variability. However, this is the first study that investigated a wide panel of sICs in both serum and plasma from healthy donors. Further studies, also in patients' cohorts, are necessary to deepen our understanding of the choice of matrix type in relation to a specific disease and correctly interpret the results. Moreover, studies that compare different anti‐coagulants for plasma collection will be necessary to better elucidate which is the best matrix for the evaluation of sIC.

## 5 Conclusion

This study highlights that serum and plasma are not always interchangeable for sICs detection, supporting the need for a standardized protocol for their measurements. Before starting a disease‐specific study on sICs, evaluations on pre‐analytical factors such as the type of tube collection would be necessary. With the exception of soluble CD80, the choice of matrix type influenced the detection of sICs.

## Author Contributions

**Veronica Buia:** methodology, investigation, data curation, formal analysis, visualization, writing – original draft, writing – review and editing. **Martina Bonacini:** conceptualization, methodology, data curation, formal analysis, supervision, funding acquisition, project administration, writing – original draft; writing – review and editing. **Cecilia Catellani** and **Alessandro Rossi:** methodology, investigation, writing – review and editing. **Francesco Muratore** and **Carlo Salvarani:** resources, writing – review and editing. **Alessandro Zerbini:** writing – original draft; writing – review snd editing. **Stefania Croci:** conceptualization, methodology, supervision, writing – original draft; writing – review and editing.

## Funding

This study was partially supported by the Azienda Unità Sanitaria Locale ‐ IRCCS di Reggio Emilia, grant: “Bando per la Valorizzazione della Ricerca Istituzionale 2021”.

## Ethics Statement

The study was approved by the local ethics committee (Comitato Etico dell'Area Vasta Emilia Nord) on 24/05/2022, protocol number 2022/0071669. The study was conducted in accordance with the Declaration of Helsinki.

## Conflicts of Interest

The authors declare no conflicts of interest.

## Supporting information


**Table S1:** Limits of detection for each sIC. The data are expressed in pg/mL.

## Data Availability

The data that support the findings of this study are available from the corresponding author upon reasonable request.
