# Supplementary material for: Matrix Type Influences the Levels of Soluble Immune Checkpoints
Source: J Clin Lab Anal. 2025 Dec 29;40(3):e70153. doi: 10.1002/jcla.70153 (PMC12888759; doi:10.1002/jcla.70153)
Supplement: Supplementary file 1 — Table S1: Limits of detection for each sIC. The data are expressed in pg/mL. [file JCLA-40-e70153-s001.docx]

**Matrix type influences the levels of soluble immune checkpoints**

**Veronica Buia^1,2^, Martina Bonacini^2*^, Cecilia Catellani^2^, Alessandro Rossi^2^, Francesco Muratore^3,4^, Carlo Salvarani^3,4^, Alessandro Zerbini^2^, Stefania Croci^2^**

1 PhD Program in Clinical and Experimental Medicine, University of Modena and Reggio Emilia, Modena, Italy

2 Unit of Clinical Immunology, Allergy and Advanced Biotechnologies, AUSL - IRCCS di Reggio Emilia, Italy

3 Unit of Rheumatology, AUSL - IRCCS di Reggio Emilia, Italy

4 University of Modena and Reggio Emilia, Department of Surgery, Medicine Dentistry and Morphological Sciences with Interest in Transplant, Modena, Italy

**SUPPLEMENTARY TABLE 1** Limits of detection for each sIC. The data are expressed in pg/ml.

| **Soluble ICs** | **Lower limit of detection** | **Upper limit of detection** |
| --- | --- | --- |
| 4-1BBL | 401.5 | 398085 |
| CD137 | 65.5 | 63463 |
| CD152 | 8.1 | 5576 |
| CD27 | 5.0 | 16515 |
| CD40 | 2.7 | 11304 |
| CD40L | 2.6 | 8612 |
| CD80 | 9.3 | 143896 |
| GITR | 21.9 | 50773 |
| GITRL | 10.9 | 9682 |
| ICOSL | 2.2 | 30568 |
| IDO | 56.5 | 15773 |
| LAG3 | 12.2 | 10559 |
| PD-1 | 27.3 | 23446 |
| PDL-1 | 0.9 | 8900 |
| PDL-2 | 12.8 | 39222 |
| TIM3 | 68.5 | 50655 |
| VISTA | 31.9 | 6967 |
